# Molecular and morphological data to facilitate future research on freshwater mussels (Bivalvia: Unionidae: Anodontinae)

**DOI:** 10.1016/j.dib.2017.12.050

**Published:** 2018-01-03

**Authors:** Chase H. Smith, Nathan A. Johnson, John M. Pfeiffer, Michael M. Gangloff

**Affiliations:** a US Geological Survey, Wetland and Aquatic Research Center, 7920 NW 71st Street, Gainesville, FL 32653, USA; b Florida Museum of Natural History, University of Florida, Gainesville, FL 32611, USA; c Biology Department, Appalachian State University, Boone, NC 28608-2027, USA

**Keywords:** Multi-locus sequence data, Linear morphometric data, *Strophitus*, Species boundaries

## Abstract

This data article presents the multi-locus DNA alignments, morphometric data, and details on specimens examined to resolve the evolutionary history of *Anodontoides* and *Strophitus*, primarily generic placement and species boundaries. We sequenced 3 loci to create our molecular matrix: *cytochrome c oxidase subunit I, NADH dehydrogenase subunit I*, and the nuclear-encoded ribosomal *internal transcribed spacer I*. Aligned sequences were used in phylogenetic analyses and to identify diagnostic nucleotides for *Strophitus pascagoulaensis*, *Strophitus radiatus*, *Strophitus sp.* cf. *pascagoulaensis*, and *Strophitus williamsi.* Linear morphometrics (i.e. maximum height, length, and width) were also implemented to further evaluate species boundaries within *Strophitus*. For further details and experimental findings, please refer to the article published in Molecular Phylogenetics and Evolution (Smith et al., 2018) [1].

**Specifications Table**

**Table:** 

Subject area	Biology
More specific subject area	Phylogenetics and Systematics
Type of data	Genomic sequences, morphometric measurements, specimen collection details, and diagnostic nucleotides for taxa previously recognized as *Anodontoides radiatus*
How data was acquired	Bidirectional sequencing on an ABI 3730, digital calipers, and phylogenetic methods
Data format	Aligned consensus gene sequences, raw measurements, analyzed diagnostic sites
Experimental factors	DNA was extracted from freshwater mussel mantle tissue
Experimental features	Molecular data was assembled and edited in Geneious v 6.1.2. Diagnostic nucleotides were identified in MEGA6.
Data source location	Samples were collected throughout the United States of America
Data accessibility	Morphological data, diagnostic nucleotides, and nucleotide alignments are presented in this article. Multi-locus sequence data are also available from GenBank: https://www.ncbi.nlm.nih.gov/genbank/

**Value of the data**

• Provided sequence data and alignments can be directly utilized in future phylogenetic studies on freshwater mussels (Bivalvia: Unionidae).
• All data are tied to museum vouchers, ensuring repeatability of results and facilitating further investigation of additional DNA loci and morphological characters on the same specimens used in our study.
• Sequence data are available to confirm species-level identification of existing museum specimens or samples collected during future field surveys.
• Molecular and morphological data provides insight on cryptic diversity within *Strophitus radiatus*, including diagnostic nucleotides that distinguish species within this complex.

## 1 Data

We present multi-locus sequence data for 97 individuals representing 18 species of North American freshwater mussels (Bivalvia: Unionidae) belonging to the subfamily Anodontinae. We also provide shell measurements (max length, width, and height) taken from 142 specimens used to evaluate morphological differences between *Strophitus pascagoulaensis*, *Strophitus radiatus*, *Strophitus sp.* cf. *pascagoulaensis*, and *Strophitus williamsi*. Our molecular matrix can be used as a tool for confirming identification of vouchered or field collected specimens. Additionally, by depositing vouchered specimens and providing full access to our DNA alignment files, morphometric measurements, and specimen collection information, our data provide a valuable resource for future research on freshwater mussel systematics, distribution, and conservation.

## 2 Experimental design, materials and methods

### 2.1 Molecular data collection

Our taxon sampling focused on North American freshwater mussels in the subfamily Anodontinae (Table 1). We sequenced two mitochondrial genes and one nuclear gene for phylogenetic analyses: the protein-coding mitochondrial genes *cytochrome c oxidase subunit I* (COI) and *NADH dehydrogenase subunit I* (NDI), and the nuclear-encoded ribosomal *internal transcribed spacer I* (ITSI). Tissue samples were preserved in 95% ethanol and DNA was isolated using a modified plate extraction protocol [2]. Primers used for polymerase chain reaction (PCR) and sequencing were: COI dgLCO-1490—GGTCAACAAATCATAAAGAYATYGG and COI dgHCO-2198—TAAACTTCAGGGTGACCAAARAAYCA [3]; NDI Leu-uurF- TGGCAGAAAAGTGCATCAGATTAAAGC and LoGlyR—CCTGCTTGGAAGGCAAGTGTACT [4]; ITSI-18S—AAAAAGCTTCCGTAGGTGAACCTGCG and ITSI-5.8 S—AGCTTGCTGCGTTCTTCATCG [5]. PCR amplifications were conducted using a 25 µl mixture of the following: distilled deionized water (9.5 µl), GoTaq GMM (12.5 µl) (Green Master Mix, Promega Corporation), primers (0.5 µl each) and DNA template (40 ng). Product was sent to the Interdisciplinary Center for Biotechnology Research at the University of Florida for bi-directional sequencing on an ABI 3730.

**Table 1:** Specimens and loci used in Smith et al. (2018) [1]. Taxon identifiers, museum catalog numbers, and Genbank accession numbers for all molecular loci included in the 3-gene concatenated dataset are provided. Museum abbreviations are as follows: UF (Florida Museum of Natural History); MMNS (Mississippi Museum of Natural Sciences); ASU (Appalachian State University).

Taxa	Catalog Number	Latitude	Longitude	COI	NDI	ISTI
*Alasmidonta triangulata*	UF441077.002	31.00521	−84.52261	MG199612	MG199739	MG199866
*Anodonta couperiana*	UF440939.011	29.060006	−81.382914	MG199596	MG199723	MG199850
*Anodonta hartfieldorum*	UF441275.038	30.8555	−87.31268	MG199623	MG199750	MG199877
*Anodonta heardi* 1	UF438379.006	30.12246	−85.14376	MG199610	MG199737	MG199864
*Anodonta heardi* 2	UF441265.012	31.74566	−85.12909	MG199622	MG199749	MG199876
*Anodonta suborbiculata*	MMNS10163	31.221928	−91.538085	MG199598	MG199725	MG199852
*Anodontoides ferussacianus* 1	–			MG199685	MG199812	MG199939
*Anodontoides ferussacianus* 2	–			MG199686	MG199813	MG199940
*Anodontoides ferussacianus* 3	–			MG199687	MG199814	MG199941
*Anodontoides ferussacianus* cf*. denigrata* 1	UF438162.001	36.61645	−84.02003	MG199637	MG199764	MG199891
*Anodontoides ferussacianus* cf*. denigrata* 2	UF438162.002	36.61645	−84.02003	MG199638	MG199765	MG199892
*Anodontoides ferussacianus* cf*. denigrata* 3	UF438162.003	36.61645	−84.02003	MG199639	MG199766	MG199893
*Anodontoides ferussacianus* cf*. denigrata* 4	UF438163.004	36.60361	−84.04476	MG199640	MG199767	MG199894
*Anodontoides ferussacianus* cf*. denigrata* 5	UF438163.005	36.60361	−84.04476	MG199641	MG199768	MG199895
*Anodontoides ferussacianus* cf*. denigrata* 6	UF438163.006	36.60361	−84.04476	MG199642	MG199769	MG199896
*Cristaria plicata*	–			KM233451	KM233451	EU580111
*Lasmigona etowaensis* 1	UF438474.003	34.25786	−85.30144	MG199611	MG199738	MG199865
*Lasmigona etowaensis* 2	UF438188.6068	33.98492	−85.08266	MG199651	MG199778	MG199905
*Pseudodontoideus connasaugaensis* 1	UF438199.001	33.72361	−85.59901	MG199662	MG199789	MG199916
*Pseudodontoideus connasaugaensis* 2	UF438199.002	33.72361	−85.59901	MG199663	MG199790	MG199917
*Pseudodontoideus connasaugaensis* 3	UF438199.003	33.72361	−85.59901	MG199664	MG199791	MG199918
*Pseudodontoideus connasaugaensis* 4	UF438199.005	33.72361	−85.59901	MG199665	MG199792	MG199919
*Pseudodontoideus subvexus* 1	UF438164.002	33.68795	−87.66486	MG199643	MG199770	MG199897
*Pseudodontoideus subvexus* 2	UF438195.6092	33.00305	−87.75356	MG199659	MG199786	MG199913
*Pseudodontoideus subvexus* 3	UF438195.6094	33.00305	−87.75356	MG199660	MG199787	MG199914
*Pseudodontoideus subvexus* 4	UF438195.6095	33.00305	−87.75356	MG199661	MG199788	MG199915
*Pseudodontoideus subvexus* 5	UF438194.6082	34.11203	−87.73093	MG199654	MG199781	MG199908
*Pseudodontoideus subvexus* 6	UF438194.6085	34.11203	−87.73093	MG199655	MG199782	MG199909
*Pseudodontoideus subvexus* 7	UF438194.6086	34.11203	−87.73093	MG199656	MG199783	MG199910
*Pseudodontoideus subvexus* 8	UF438194.6090	34.11203	−87.73093	MG199657	MG199784	MG199911
*Pseudodontoideus subvexus* 9	UF438194.6091	34.11203	−87.73093	MG199658	MG199785	MG199912
*Pseudodontoideus subvexus* 10	UF438201.6121	32.36612	−88.41522	MG199671	MG199798	MG199925
*Pyganodon grandis* 1	UF441315.042	31.35521	−85.08589	MG199624	MG199751	MG199878
*Pyganodon grandis* 2	UF438033.043	30.85467	−87.31101	MG199625	MG199752	MG199879
*Strophitus pascagoulaensis* 1	UF438202.6129	32.26286	−88.56979	MG199672	MG199799	MG199926
*Strophitus pascagoulaensis* 2	UF438202.6130	32.26286	−88.56979	MG199673	MG199800	MG199927
*Strophitus pascagoulaensis* 3	UF439322	32.26286	−88.56979	MG199674	MG199801	MG199928
*Strophitus pascagoulaensis* 4	UF438202.6123	32.26286	−88.56979	MG199711	MG199838	–
*Strophitus pascagoulaensis* 5	UF438202.6124	32.26286	−88.56979	MG199712	MG199839	–
*Strophitus pascagoulaensis* 6	UF438202.6125	32.26286	−88.56979	MG199713	MG199840	–
*Strophitus pascagoulaensis* 7	UF438202.6126	32.26286	−88.56979	MG199714	MG199841	–
*Strophitus pascagoulaensis* 8	UF438202.6127	32.26286	−88.56979	MG199715	MG199842	–
*Strophitus pascagoulaensis* 9	UF438202.6128	32.26286	−88.56979	MG199716	MG199843	–
*Strophitus pascagoulaensis* 10	UF438202.6131	32.26286	−88.56979	MG199717	MG199844	–
*Strophitus radiatus* 1	UF438206.6139	34.818	−89.05283	MG199676	MG199803	MG199930
*Strophitus radiatus* 2	UF438206.6140	34.818	−89.05283	MG199677	MG199804	MG199931
*Strophitus radiatus* 3	UF438880.011	32.949566	−87.96215	MG199608	MG199735	MG199862
*Strophitus radiatus* 4	UF438880.012	32.949566	−87.96215	MG199609	MG199736	MG199863
*Strophitus radiatus* 5	UF438200.6108	32.36612	−88.41522	MG199666	MG199793	MG199920
*Strophitus radiatus* 6	UF438200.6111	32.36612	−88.41522	MG199667	MG199794	MG199921
*Strophitus radiatus* 7	UF438200.6113	32.36612	−88.41522	MG199668	MG199795	MG199922
*Strophitus radiatus* 8	UF438200.6119	32.36612	−88.41522	MG199669	MG199796	MG199923
*Strophitus radiatus* 9	UF438200.6120	32.36612	−88.41522	MG199670	MG199797	MG199924
*Strophitus radiatus* 10	UF438160.032	34.11203	−87.73093	MG199632	MG199759	MG199886
*Strophitus radiatus* 11	UF438795.005	33.76028	−87.6104	MG199599	MG199726	MG199853
*Strophitus radiatus* 12	UF438167.043	33.00305	−87.75356	MG199647	MG199774	MG199901
*Strophitus radiatus* 13	UF438167.044	33.00305	−87.75356	MG199648	MG199775	MG199902
*Strophitus radiatus* 14	UF438167.046	33.00305	−87.75356	MG199649	MG199776	MG199903
*Strophitus radiatus* 15	UF438167.048	33.00305	−87.75356	MG199650	MG199777	MG199904
*Strophitus radiatus* 16	UF438165.041	32.01874	−85.29579	MG199646	MG199773	MG199900
*Strophitus radiatus* 17	UF438207.6141	32.3789	−85.1818	MG199678	MG199805	MG199932
*Strophitus radiatus* 18	UF438165.039	32.01874	−85.29579	MG199644	MG199771	MG199898
*Strophitus radiatus* 19	UF438165.040	32.01874	−85.29579	MG199645	MG199772	MG199899
*Strophitus radiatus* 20	UF441078.006	31.069118	−85.405	MG199601	MG199728	MG199855
*Strophitus radiatus* 21	UF441078.007	31.069118	−85.405	MG199602	MG199729	MG199856
*Strophitus radiatus* 22	UF441078.008	31.069118	−85.405	MG199603	MG199730	MG199857
*Strophitus radiatus* 23	UF441131.009	31.074716	−85.218983	MG199604	MG199731	MG199858
*Strophitus radiatus* 24	UF438197.010	32.20303341	−84.41530078	MG199605	MG199732	MG199859
*Strophitus radiatus* 25	ASU5224.9857	31.92777	−87.01426	MG199689	MG199816	MG199943
*Strophitus radiatus* 26	ASU5224.9856	31.92777	−87.01426	MG199721	MG199848	–
*Strophitus radiatus* 27	ASU5224.9858	31.92777	−87.01426	MG199722	MG199849	–
*Strophitus radiatus* 28	UF438167.042	33.00305	−87.75356	MG199698	MG199825	–
*Strophitus radiatus* 29	UF438167.045	33.00305	−87.75356	MG199699	MG199826	–
*Strophitus radiatus* 30	UF438167.047	33.00305	−87.75356	MG199700	MG199827	–
*Strophitus radiatus* 31	UF438167.049	33.00305	−87.75356	MG199701	MG199828	–
*Strophitus radiatus* 32	UF438167.050	33.00305	−87.75356	MG199702	MG199829	–
*Strophitus radiatus* 33	UF438200.6109	32.36612	−88.41522	MG199703	MG199830	–
*Strophitus radiatus* 34	UF438200.6110	32.36612	−88.41522	MG199704	MG199831	–
*Strophitus radiatus* 35	UF438200.6112	32.36612	−88.41522	MG199705	MG199832	–
*Strophitus radiatus* 36	UF438200.6114	32.36612	−88.41522	MG199706	MG199833	–
*Strophitus radiatus* 37	UF438200.6115	32.36612	−88.41522	MG199707	MG199834	–
*Strophitus radiatus* 38	UF438200.6116	32.36612	−88.41522	MG199708	MG199835	–
*Strophitus radiatus* 39	UF438200.6117	32.36612	−88.41522	MG199709	MG199836	–
*Strophitus radiatus* 40	UF438200.6118	32.36612	−88.41522	MG199710	MG199837	–
*Strophitus radiatus* 41	UF438210.6145	34.75669	−89.14737	MG199719	MG199846	–
*Strophitus radiatus* 42	UF441279.015	31.105616667	−85.25436667	MG199692	MG199819	–
*Strophitus sp.* cf*. pascagoulaensis* 1	UF438877	32.10864	−90.21003	MG199600	MG199727	MG199854
*Strophitus sp.* cf*. pascagoulaensis* 2	UF438205.6137	31.23189	−90.86655	MG199675	MG199802	MG199929
*Strophitus sp.* cf*. pascagoulaensis* 3	UF438205.6138	31.23189	−90.86655	MG199691	MG199818	MG199945
*Strophitus sp.* cf*. pascagoulaensis* 4	UF438205.6136	31.23189	−90.86655	MG199718	MG199845	–
*Strophitus undulatus* 1	UF438191.5063	30.65781	−99.32435	MG199626	MG199753	MG199880
*Strophitus undulatus* 2	UF438744.6650	30.658673	−99.3245	MG199681	MG199808	MG199935
*Strophitus undulatus* 3	UF438744.6651	30.658673	−99.3245	MG199682	MG199809	MG199936
*Strophitus undulatus* 4	UF438744.6653	30.658673	−99.3245	MG199683	MG199810	MG199937
*Strophitus undulatus* 5	UF438744.6654	30.658673	−99.3245	MG199684	MG199811	MG199938
*Strophitus undulatus* 6	UF438193.6077	36.13527	−83.20188	MG199652	MG199779	MG199906
*Strophitus undulatus* 7	UF438193.6078	36.13527	−83.20188	MG199653	MG199780	MG199907
*Strophitus undulatus* 8	ASU5186.9855	35.688	−79.94872	MG199688	MG199815	MG199942
*Strophitus undulatus* 9	ASU5185.9859	35.9367	−78.149408	MG199690	MG199817	MG199944
*Strophitus williamsi* 1	UF438158.024	31.58155	−86.91755	MG199627	MG199754	MG199881
*Strophitus williamsi* 2	UF438158.026	31.58155	−86.91755	MG199628	MG199755	MG199882
*Strophitus williamsi* 3	UF438158.027	31.58155	−86.91755	MG199629	MG199756	MG199883
*Strophitus williamsi* 4	UF438158.028	31.58155	−86.91755	MG199630	MG199757	MG199884
*Strophitus williamsi* 5	UF438159.031	31.30133	−87.01215	MG199631	MG199758	MG199885
*Strophitus williamsi* 6	UF438158.6464	31.58155	−86.91755	MG199679	MG199806	MG199933
*Strophitus williamsi* 7	UF438158.6465	31.58155	−86.91755	MG199680	MG199807	MG199934
*Strophitus williamsi* 8	UF438713.004	31.043	−86.1013	MG199597	MG199724	MG199851
*Strophitus williamsi* 9	UF438161.033	31.10979	−86.14611	MG199633	MG199760	MG199887
*Strophitus williamsi* 10	UF438161.034	31.10979	−86.14611	MG199634	MG199761	MG199888
*Strophitus williamsi* 11	UF438161.035	31.10979	−86.14611	MG199635	MG199762	MG199889
*Strophitus williamsi* 12	UF438161.038	31.10979	−86.14611	MG199636	MG199763	MG199890
*Strophitus williamsi* 13	UF441102.013	30.61419	−86.01275	MG199613	MG199740	MG199867
*Strophitus williamsi* 14	UF441102.014	30.61419	−86.01275	MG199614	MG199741	MG199868
*Strophitus williamsi* 15	UF441102.015	30.61419	−86.01275	MG199615	MG199742	MG199869
*Strophitus williamsi* 16	UF441102.016	30.61419	−86.01275	MG199616	MG199743	MG199870
*Strophitus williamsi* 17	UF441102.017	30.61419	−86.01275	MG199617	MG199744	MG199871
*Strophitus williamsi* 18	UF439319	30.61419	−86.01275	MG199618	MG199745	MG199872
*Strophitus williamsi* 19	UF441102.019	30.61419	−86.01275	MG199619	MG199746	MG199873
*Strophitus williamsi* 20	UF441102.020	30.61419	−86.01275	MG199620	MG199747	MG199874
*Strophitus williamsi* 21	UF441102.021	30.61419	−86.01275	MG199621	MG199748	MG199875
*Strophitus williamsi* 22	UF438161.036	31.10979	−86.14611	MG199696	MG199823	–
*Strophitus williamsi* 23	UF438161.037	31.10979	−86.14611	MG199697	MG199824	–
*Strophitus williamsi* 24	UF438158.025	31.58155	−86.91755	MG199693	MG199820	–
*Strophitus williamsi* 25	UF438158.029	31.58155	−86.91755	MG199694	MG199821	–
*Strophitus williamsi* 26	UF438158.030	31.58155	−86.91755	MG199695	MG199822	–
*Strophitus williamsi* 27	UF438158.6463	31.58155	−86.91755	MG199720	MG199847	–
*Utterbackia peggyae* 1	UF441117.040	30.48789	−84.39746	MG199606	MG199733	MG199860
*Utterbackia peggyae* 2	UF441117.041	30.48789	−84.39746	MG199607	MG199734	MG199861

### 2.2 Molecular data processing

Chromatograms were assembled and edited in Geneious v 6.1.2 [6] and consensus sequences were aligned in Mesquite v 3.1.0 [7] using MAFFT v 7.299 [8]. The mtDNA protein coding genes COI and NDI were aligned then translated into amino acids to ensure absence of gaps and stop codons. The ITSI alignment was performed using the default parameters in MAFFT and minor adjustments were made by eye where necessary. Independent alignment files (phylip format) for COI, NDI, and ITSI are available in supplemental information (Supplemental_File_1_All_COI.phy; Supplemental_File_2_All_NDI.phy; Supplemental_File_3_All_ITSI.phy). Diagnostic nucleotides within *S. pascagoulaensis*, *S. radiatus*, *S. sp.* cf. *pascagoulaensis*, and *S. williamsi* were identified using MEGA6 [9]. COI, NDI, and ITSI were independently analyzed (including redundant haplotypes) and groups were determined by species: *S. pascagoulaensis*, *S. radiatus*, *S. sp.* cf. *pascagoulaensis*, and *S. williamsi*. We used the mitochondrial genome sequence of *Cristaria plicata* (GenBank Accession: KM233451) to determine nucleotide positions for COI and NDI. Base pair positions at ITSI were determined by using an alignment consisting of only *A. radiatus* (Supplemental_File_4_A_radiatus_ITSI.phy). We used the sequence of our only *A. radiatus* from the Pearl River as a reference (Genbank accession number MG199854). Diagnostic nucleotides are provided in Table 2.

**Table 2:** Diagnostic nucleotides referenced in Smith et al. (2018) [1] for *Strophitus pascagoulaensis*, *Strophitus radiatus*, *Strophitus sp.* cf. *pascagoulaensis,* and *Strophitus williamsi* with indication of whether the substitution changed the amino acid translation.

Gene	Position	*S. pascagoulaensis*	*S. radiatus*	*S. sp* cf. *pascagoulaensis*	*S. williamsi*	Amino Acid Change (Y/N)
CO1	52	A	A	C	A	N
CO1	144	C	C	T	C	N
CO1	222	A	G	G	G	N
CO1	261	G	G	G	A	N
CO1	267	A	G	G	G	N
CO1	276	T	T	C	T	N
CO1	291	G	G	A	G	N
CO1	300	A	A	A	G	N
CO1	336	T	T	G	T	N
CO1	342	G	A	G	G	N
CO1	363	T	T	T	G	N
CO1	375	A	G	G	G	N
CO1	408	T	T	C	T	N
CO1	411	G	G	G	A	N
CO1	429	C	T	T	T	N
CO1	459	T	T	G	T	N
CO1	471	C	T	T	T	N
CO1	498	G	A	A	A	N
CO1	540	A	A	G	A	N
CO1	568	A	G	G	G	Y
CO1	570	A	G	A	A	N
CO1	571	C	T	T	T	N
CO1	573	A	G	A	A	N
CO1	576	A	A	G	A	N
CO1	579	T	T	C	T	N
CO1	612	A	A	A	G	N
CO1	627	T	C	C	C	N
CO1	657	G	T	T	T	N
CO1	663	G	G/T	G	A	N
ND1	10	C	T	C	C	Y
ND1	23	C	C	T	C	Y
ND1	24	T	T	T	C	N
ND1	25	C	C	A	C	Y
ND1	26	T	T	C	T	Y
ND1	27	T	T	C	T	N
ND1	39	C	C	T	C	N
ND1	40	T	T	C	T	N
ND1	63	T	T	C	T	N
ND1	90	T	T	T	C	N
ND1	120	C	T	C	C	N
ND1	123	G	G	G	A	N
ND1	162	C	C	T	C	N
ND1	177	C	T	C	C	N
ND1	188	T	C	T	T	Y
ND1	231	G	T	G	A	N
ND1	264	A	A	G	A	N
ND1	273	C	C	C	T	N
ND1	297	A	A	G	A	N
ND1	304	A	C/T	C	C	Y
ND1	318	G	A	A	A	N
ND1	326	G	G	G	C	Y
ND1	327	C	T	T	T	N
ND1	370	G	G	A	G	Y
ND1	396	G	G	A	G	N
ND1	411	C	T	T	T	N
ND1	423	A	A	A	G	N
ND1	432	C	T	T	T	N
ND1	466	T	C	C	C	N
ND1	468	A	A	G	A	N
ND1	471	T	C/A	C	C	N
ND1	477	A	A	T	A	N
ND1	486	G	A	A	A	N
ND1	489	C	C	C	T	N
ND1	532	G	A	G	G	Y
ND1	555	G	A	A	A	N
ND1	565	A	G	G	G	Y
ND1	576	C	T	T	T	N
ND1	612	G	G	A	G	N
ND1	642	C	C	T	C	N
ND1	700	A	T	T	T	Y
ND1	745	G	A	A	A	Y
ND1	749	T	C	T	T	Y
ND1	750	A	G	G	G	N
ND1	766	A	A	G	A	Y
ND1	768	T	T	T	C	N
ND1	770	C	C	T	C	Y
ITS1	52	A	T/-	–	T/-	n/a
ITS1	53	A	A	–	A	n/a
ITS1	56	C	A/T	G	A	n/a
ITS1	406	G	A	A	A	n/a

### 2.3 Morphological data collection

We conducted linear morphometrics of external shell dimensions for all *S. pascagoulaensis*, *S. radiatus*, *S. sp.* cf. *pascagoulaensis*, and *S. williamsi* specimens used in genetic analyses along with additional museum specimens from focal drainages. Three measurements were taken for morphological analyses: maximum length (anterior to posterior), height (dorsal to ventral), and width (right to left valve) to the nearest 0.01 mm using digital calipers. One person conducted all measurements, and height and width were measured at the posterior end of the hinge ligament for consistency. Raw measurements are provided in Table 3.

**Table 3:** Morphological data referenced in Smith et al. (2018) [1] for all *Strophitus pascagoulaensis*, *Strophitus radiatus*, *Strophitus sp.* cf. *pascagoulaensis,* and *Strophitus williamsi*.

Taxa	Catalog Number	Latitude	Longitude	Length (mm)	Height (mm)	Width (mm)
*Strophitus pascagoulaensis*	MMNS10064.1	32.23872912	−88.57336007	45.97	27.02	15.31
*Strophitus pascagoulaensis*	MMNS10090.1	31.96429574	−88.51815688	39.98	23.79	13.54
*Strophitus pascagoulaensis*	MMNS10090.2	31.96429574	−88.51815688	37.65	21.48	13.95
*Strophitus pascagoulaensis*	MMNS10090.3	31.96429574	−88.51815688	52.37	29.38	22.01
*Strophitus pascagoulaensis*	MMNS10090.4	31.96429574	−88.51815688	51.63	28.59	19.48
*Strophitus pascagoulaensis*	MMNS6457.1	31.52414348	−89.65462583	56.51	33.11	19.12
*Strophitus pascagoulaensis*	MMNS7545.1	31.3713172	−89.1190562	39.42	22.55	13.39
*Strophitus pascagoulaensis*	UF438202.6122	32.26286	−88.56979	56.39	32.96	21.2
*Strophitus pascagoulaensis*	UF438202.6123	32.26286	−88.56979	58.1	30.37	21.79
*Strophitus pascagoulaensis*	UF438202.6124	32.26286	−88.56979	56.71	32.08	22.53
*Strophitus pascagoulaensis*	UF438202.6125	32.26286	−88.56979	56.88	32.68	22.39
*Strophitus pascagoulaensis*	UF438202.6126	32.26286	−88.56979	45.82	28.12	17.95
*Strophitus pascagoulaensis*	UF438202.6127	32.26286	−88.56979	43.64	25.47	15.4
*Strophitus pascagoulaensis*	UF438202.6128	32.26286	−88.56979	42.47	26.66	14.58
*Strophitus pascagoulaensis*	UF438202.6129	32.26286	−88.56979	42.7	24.77	14.14
*Strophitus pascagoulaensis*	UF438202.6130	32.26286	−88.56979	39.73	23.99	14.12
*Strophitus pascagoulaensis*	UF438202.6131	32.26286	−88.56979	38.66	24.21	13.67
*Strophitus pascagoulaensis*	UF439322	32.26286	−88.56979	38.36	23.31	14.83
*Strophitus pascagoulaensis*	UF438202.6133	32.26286	−88.56979	33.62	20.44	12.07
*Strophitus radiatus*	ASU5224.9856	31.92777	−87.01426	45.07	24.91	14.72
*Strophitus radiatus*	ASU5224.9857	31.92777	−87.01426	45.7	27.45	14.85
*Strophitus radiatus*	ASU5224.9858	31.92777	−87.01426	47.91	26.74	16.06
*Strophitus radiatus*	MMNS10819.1	34.8572182	−89.80544458	84.6	47.52	26.76
*Strophitus radiatus*	MMNS10819.2	34.8572182	−89.80544458	74.48	41.53	25.57
*Strophitus radiatus*	MMNS5833.1	34.43668209	−89.36627264	74.65	38.12	24.16
*Strophitus radiatus*	MMNS8933.1	34.48095713	−89.20274397	54.27	28.44	18.32
*Strophitus radiatus*	MMNS9019.1	34.51633055	−89.18345921	65.17	35.66	21.95
*Strophitus radiatus*	UF438160.032	34.11203	−87.73093	39.7	22.39	13.1
*Strophitus radiatus*	UF438165.039	32.01874	−85.29579	48.49	26.31	16.29
*Strophitus radiatus*	UF438165.040	32.01874	−85.29579	58.66	30.49	21.38
*Strophitus radiatus*	UF438165.041	32.01874	−85.29579	45.28	24.63	15.4
*Strophitus radiatus*	UF438167.042	33.00305	−87.75356	61.97	33.42	22.95
*Strophitus radiatus*	UF438167.043	33.00305	−87.75356	58.71	30.97	19.92
*Strophitus radiatus*	UF438167.044	33.00305	−87.75356	59.3	31.74	21.63
*Strophitus radiatus*	UF438167.045	33.00305	−87.75356	64.59	32.14	23.11
*Strophitus radiatus*	UF438167.046	33.00305	−87.75356	62.98	34.8	23.3
*Strophitus radiatus*	UF438167.047	33.00305	−87.75356	48.22	27.35	14.8
*Strophitus radiatus*	UF438167.048	33.00305	−87.75356	51.13	28.1	18.16
*Strophitus radiatus*	UF438167.049	33.00305	−87.75356	56.62	30.64	20.34
*Strophitus radiatus*	UF438167.050	33.00305	−87.75356	51.37	27.86	19.49
*Strophitus radiatus*	UF438197.010	32.20303341	−84.41530078	58.91	28.14	21.91
*Strophitus radiatus*	UF438200.6108	32.36612	−88.41522	53.56	31.97	18.57
*Strophitus radiatus*	UF438200.6109	32.36612	−88.41522	50.95	28.97	15.82
*Strophitus radiatus*	UF438200.6110	32.36612	−88.41522	47.85	27.23	15.52
*Strophitus radiatus*	UF438200.6111	32.36612	−88.41522	45.3	26.92	16.85
*Strophitus radiatus*	UF438200.6112	32.36612	−88.41522	46.14	26.65	15.43
*Strophitus radiatus*	UF438200.6113	32.36612	−88.41522	39.12	23.26	13.44
*Strophitus radiatus*	UF438200.6114	32.36612	−88.41522	39.01	22.67	13.37
*Strophitus radiatus*	UF438200.6115	32.36612	−88.41522	36.55	20.56	11.99
*Strophitus radiatus*	UF438200.6116	32.36612	−88.41522	36.93	21.46	11.85
*Strophitus radiatus*	UF438200.6117	32.36612	−88.41522	33.72	20.56	11.57
*Strophitus radiatus*	UF438200.6118	32.36612	−88.41522	33.36	19.64	11.32
*Strophitus radiatus*	UF438200.6119	32.36612	−88.41522	32.83	19.46	11.02
*Strophitus radiatus*	UF438200.6120	32.36612	−88.41522	32.14	19.05	11.28
*Strophitus radiatus*	UF438206.6139	34.818	−89.05283	51.7	25.93	15.62
*Strophitus radiatus*	UF438206.6140	34.818	−89.05283	41.14	21.63	12.82
*Strophitus radiatus*	UF438207.6141	32.3789	−85.1818	40.6	23.31	14.5
*Strophitus radiatus*	UF438210.6145	34.75669	−89.14737	33.62	18.51	10.1
*Strophitus radiatus*	UF438252.6277	30.43084	−85.17185	70.82	38.89	28.12
*Strophitus radiatus*	UF438795.005	33.76028	−87.6104	29.77	17.04	10.8
*Strophitus radiatus*	UF438880.011	32.949566	−87.96215	48.09	29.16	16.88
*Strophitus radiatus*	UF438880.012	32.949566	−87.96215	48.34	26.07	16.24
*Strophitus radiatus*	UF438993.8984	31.823788	−87.386155	37.47	21.35	12.68
*Strophitus radiatus*	UF438994.8986	32.875918	−87.927079	51.82	29.53	20.1
*Strophitus radiatus*	UF441078.006	31.069118	−85.405	62.1	36.08	23.11
*Strophitus radiatus*	UF441078.007	31.069118	−85.405	62.37	34.16	24.77
*Strophitus radiatus*	UF441078.008	31.069118	−85.405	66.54	36.32	26.68
*Strophitus radiatus*	UF441131.009	31.074716	−85.218983	51	26.73	19.47
*Strophitus radiatus*	UF441319.023	31.83791	−86.90898	40.55	23.6	12.44
*Strophitus sp.* cf*. pascagoulaensis*	MMNS10113	32.85110816	−89.64942898	28.65	17.18	10.17
*Strophitus sp.* cf*. pascagoulaensis*	MMNS13006.1	30.79567	−90.45228	71.66	36.02	23.29
*Strophitus sp.* cf*. pascagoulaensis*	MMNS2329.1	32.19795718	−90.16632856	77.76	41.37	28.15
*Strophitus sp.* cf*. pascagoulaensis*	MMNS2377.10	32.19795718	−90.16632856	41.08	23.45	14.22
*Strophitus sp.* cf*. pascagoulaensis*	MMNS2377.11	32.19795718	−90.16632856	43.42	22.68	15.38
*Strophitus sp.* cf*. pascagoulaensis*	MMNS2377.14	32.19795718	−90.16632856	43.46	24.34	13.96
*Strophitus sp.* cf*. pascagoulaensis*	MMNS2377.15	32.19795718	−90.16632856	43.84	24.92	15.78
*Strophitus sp.* cf*. pascagoulaensis*	MMNS2377.16	32.19795718	−90.16632856	44.64	24.53	16.6
*Strophitus sp.* cf*. pascagoulaensis*	MMNS2377.18	32.19795718	−90.16632856	44.12	24.79	14.98
*Strophitus sp.* cf*. pascagoulaensis*	MMNS2377.2	32.19795718	−90.16632856	31.16	17.99	10.1
*Strophitus sp.* cf*. pascagoulaensis*	MMNS2377.20	32.19795718	−90.16632856	45.17	26.12	15.28
*Strophitus sp.* cf*. pascagoulaensis*	MMNS2377.21	32.19795718	−90.16632856	46.46	27.39	18.06
*Strophitus sp.* cf*. pascagoulaensis*	MMNS2377.22	32.19795718	−90.16632856	47.76	28.12	15.71
*Strophitus sp.* cf*. pascagoulaensis*	MMNS2377.25	32.19795718	−90.16632856	49.19	27.76	17.83
*Strophitus sp.* cf*. pascagoulaensis*	MMNS2377.27	32.19795718	−90.16632856	48.98	28.12	16.26
*Strophitus sp.* cf*. pascagoulaensis*	MMNS2377.28	32.19795718	−90.16632856	81.39	43.46	29.18
*Strophitus sp.* cf*. pascagoulaensis*	MMNS2377.4	32.19795718	−90.16632856	36.99	21.13	12.5
*Strophitus sp.* cf*. pascagoulaensis*	MMNS2377.6	32.19795718	−90.16632856	39.07	22.73	15.5
*Strophitus sp.* cf*. pascagoulaensis*	MMNS2377.8	32.19795718	−90.16632856	41.09	23.77	15.54
*Strophitus sp.* cf*. pascagoulaensis*	MMNS2657.1	31.01063379	−90.46129141	42.81	24.8	14.85
*Strophitus sp.* cf*. pascagoulaensis*	MMNS2657.3	31.01063379	−90.46129141	60.14	33.32	23.85
*Strophitus sp.* cf*. pascagoulaensis*	MMNS2657.4	31.01063379	−90.46129141	63.49	33.23	22.54
*Strophitus sp.* cf*. pascagoulaensis*	MMNS2657.5	31.01063379	−90.46129141	70.66	37.3	27.29
*Strophitus sp.* cf*. pascagoulaensis*	MMNS4722.1	32.19795718	−90.16632856	88.81	47.9	32.49
*Strophitus sp.* cf*. pascagoulaensis*	MMNS4722.2	32.19795718	−90.16632856	52.2	29.31	18.72
*Strophitus sp.* cf*. pascagoulaensis*	MMNS6267.1	31.33876638	−90.74536506	74.31	38.37	29.44
*Strophitus sp.* cf*. pascagoulaensis*	MMNS6390.1	31.28101243	−90.77000317	70.58	38.52	23.32
*Strophitus sp.* cf*. pascagoulaensis*	MMNS6390.4	31.28101243	−90.77000317	67.11	36.49	25.27
*Strophitus sp.* cf*. pascagoulaensis*	MMNS6411.1	31.04648752	−90.63409686	42.82	25.74	12.84
*Strophitus sp.* cf*. pascagoulaensis*	MMNS6411.2	31.04648752	−90.63409686	47.87	27.72	17.28
*Strophitus sp.* cf*. pascagoulaensis*	MMNS6460.1	32.19795718	−90.16632856	27.4	16.64	8.58
*Strophitus sp.* cf*. pascagoulaensis*	MMNS6460.2	32.19795718	−90.16632856	28.07	16.85	8.87
*Strophitus sp.* cf*. pascagoulaensis*	MMNS6773.1	31.28071491	−90.77001018	74.46	40.33	29.45
*Strophitus sp.* cf*. pascagoulaensis*	MMNS6807.1	31.23495345	−90.06397456	56.24	30.84	19.51
*Strophitus sp.* cf*. pascagoulaensis*	MMNS6807.3	31.23495345	−90.06397456	51.66	27.63	19.25
*Strophitus sp.* cf*. pascagoulaensis*	MMNS7596.1	32.19977	−90.16641	48.04	26.1	15.43
*Strophitus sp.* cf*. pascagoulaensis*	MNNS2377.7	32.19795718	−90.16632856	40.35	22.9	13.62
*Strophitus sp.* cf*. pascagoulaensis*	UF438205.6136	31.23189	−90.86655	60.51	33.7	21.23
*Strophitus sp.* cf*. pascagoulaensis*	UF438205.6137	31.23189	−90.86655	50.52	30.28	17.16
*Strophitus sp.* cf*. pascagoulaensis*	UF438205.6138	31.23189	−90.86655	50.87	29.58	20.23
*Strophitus sp.* cf*. pascagoulaensis*	UF438877.061	32.10864	−90.21003	39.46	23.34	14.37
*Strophitus williamsi*	UF438158.024	31.58155	−86.91755	49.8	29.34	18
*Strophitus williamsi*	UF438158.025	31.58155	−86.91755	33.4	19.49	11.59
*Strophitus williamsi*	UF438158.026	31.58155	−86.91755	38.19	21.44	13.55
*Strophitus williamsi*	UF438158.027	31.58155	−86.91755	35	21.26	12.55
*Strophitus williamsi*	UF438158.028	31.58155	−86.91755	39.41	23.09	15.14
*Strophitus williamsi*	UF438158.029	31.58155	−86.91755	35.82	22.93	12.42
*Strophitus williamsi*	UF438158.030	31.58155	−86.91755	27.99	17.1	10.6
*Strophitus williamsi*	UF438158.6463	31.58155	−86.91755	35.95	21.09	12.57
*Strophitus williamsi*	UF438158.6464	31.58155	−86.91755	38.44	22.43	14.83
*Strophitus williamsi*	UF438158.6465	31.58155	−86.91755	37.82	22.82	14.05
*Strophitus williamsi*	UF438159.031	31.30133	−87.01215	29.98	17.57	10.52
*Strophitus williamsi*	UF438161.033	31.10979	−86.14611	46.36	28.38	18.02
*Strophitus williamsi*	UF438161.034	31.10979	−86.14611	40.91	24.38	15.31
*Strophitus williamsi*	UF438161.035	31.10979	−86.14611	45.55	26.94	17.35
*Strophitus williamsi*	UF438161.036	31.10979	−86.14611	46.57	27.31	17.45
*Strophitus williamsi*	UF438161.037	31.10979	−86.14611	38.56	23.65	14.54
*Strophitus williamsi*	UF438161.038	31.10979	−86.14611	43.86	24.94	15.58
*Strophitus williamsi*	UF438713.004	31.043	−86.1013	32.19	19.05	11.65
*Strophitus williamsi*	UF438984.8942	30.62409	−85.98934	49.79	27.19	18.88
*Strophitus williamsi*	UF438984.8943	30.62409	−85.98934	42.07	24.78	15.69
*Strophitus williamsi*	UF438985.8944	30.62184	−86.06598	50.11	27.03	18.46
*Strophitus williamsi*	UF438995.8985	31.30198	−87.01244	40.73	23.97	16.5
*Strophitus williamsi*	UF441102.013	30.61419	−86.01275	53.56	27.67	20.05
*Strophitus williamsi*	UF441102.014	30.61419	−86.01275	51.17	26.69	17.94
*Strophitus williamsi*	UF441102.015	30.61419	−86.01275	50.12	27.4	18.45
*Strophitus williamsi*	UF441102.016	30.61419	−86.01275	47.03	26.87	17.76
*Strophitus williamsi*	UF441102.017	30.61419	−86.01275	46.62	25.41	17.49
*Strophitus williamsi*	UF439319	30.61419	−86.01275	41.53	23.26	15.78
*Strophitus williamsi*	UF441102.019	30.61419	−86.01275	44.76	25.88	16.6
*Strophitus williamsi*	UF441102.020	30.61419	−86.01275	44.04	24.9	16.66
*Strophitus williamsi*	UF441102.021	30.61419	−86.01275	41.72	24.05	15.43
*Strophitus williamsi*	UF441293.022	31.780642	−86.223281	53.22	30.69	22.4
