# Supplementary material for: Molecular and morphological data to facilitate future research on freshwater mussels (Bivalvia: Unionidae: Anodontinae)
Source: Data Brief. 2018 Jan 3;17:95–104. doi: 10.1016/j.dib.2017.12.050 (PMC5988452; doi:10.1016/j.dib.2017.12.050)
Supplement: Supplementary file 1 — Supplementary material [file mmc1.docx]

Declarations of interest: none
